# A minute ostracod (Crustacea: Cytheromatidae) from the Miocene Solimões Formation (western Amazonia, Brazil): evidence for marine incursions?

**DOI:** 10.1080/14772019.2015.1078850

**Published:** 2015-10-02

**Authors:** Martin Gross, Maria Ines F. Ramos, Werner E. Piller

**Affiliations:** ^a^ Department for Geology and Palaeontology, Universalmuseum Joanneum, Weinzöttlstrasse 16, 8045Graz, Austria; ^b^ Coordenação de Ciências da Terra e Ecologia, Museu Paraense Emílio Goeldi, Avenida Perimetral, 1901, Terra Firme, Belém-PA66077-830, Brazil; ^c^ Institute of Earth Sciences, University of Graz, NAWI Graz, Heinrichstrasse 26, 8010Graz, Austria

**Keywords:** *Pellucistoma*, biogeography, palaeogeography, palaeoecology, dispersal mechanisms, freshwater adaptation

## Abstract

A huge wetland (the ‘Pebas system’) covered western Amazonia during the Miocene, hosting a highly diverse and endemic aquatic fauna. One of the most contentious issues concerns the existence, potential pathways and effects of marine incursions on this ecosystem. Palaeontological evidences (body fossils) are rare. The finding of a new, presumably marine ostracod species (*Pellucistoma curupira* sp. nov.) in the upper middle Miocene Solimões Formation initiated a taxonomic, ecological and biogeographical review of the genus *Pellucistoma*. We demonstrate that this marine (sublittoral, euhaline), subtropical–tropical taxon is biogeographically confined to the Americas. The biogeographical distribution of *Pellucistoma* largely depends on geographical, thermal and osmotic barriers (e.g. land bridges, deep and/or cold waters, sea currents, salinity). We assume an Oligocene/early Miocene, Caribbean origin for *Pellucistoma* and outline the dispersal of hitherto known species up to the Holocene. *Pellucistoma curupira* sp. nov. is dwarfed in comparison to all other species of this genus and extremely thin-shelled. This is probably related to poorly oxygenated waters and, in particular, to strongly reduced salinity. The associated ostracod fauna (dominated by the eurypotent *Cyprideis* and a few, also stunted ostracods of possibly marine ancestry) supports this claim. Geochemical analyses (δ^18^O, δ^13^C) on co-occurring ostracod valves (*Cyprideis* spp.) yielded very light values, indicative of a freshwater setting. These observations point to a successful adaptation of *P. curupira* sp. nov. to freshwater conditions and therefore do not signify the presence of marine water. *Pellucistoma curupira* sp. nov. shows closest affinities to Caribbean species. We hypothesize that *Pellucistoma* reached northern South America (Llanos Basin) during marine incursions in the early Miocene. While larger animals of marine origin (e.g. fishes, dolphins, manatees) migrated actively into the Pebas wetland via fluvial connections, small biota (e.g. *P. curupira* sp. nov.) were phoretically freighted and developed freshwater tolerance over long timescales.

http://zoobank.org/urn:lsid:zoobank.org:pub:886C6476-393D-4323-8C0E-06BB8BD02FD9

## Introduction

During the Miocene epoch, an enormous wetland shaped western Amazonia's landscapes and biota (the ‘Pebas system’; for comprehensive synopses see Hoorn & Wesselingh 2010; Hoorn *et al*. 2010a). The general understanding of this unique ecosystem has significantly improved in the last two decades. In detail, however, its nature remains controversial and disputed (e.g. ‘mega-lake’, Wesselingh *et al*. 2002; ‘mega-wetland’, Hoorn *et al*. 2010b; ‘mega-fan’, Latrubesse *et al*. 2010; Wilkinson *et al*. 2010).

In particular, the existence, chronology, spatial extent and potential sources of marine interferences continue to be a heavily (and sometimes paradigmatically) discussed subject of western Amazonia's past. A plethora of sedimentological, palaeontological and geochemical indications were depicted to infer marine influences (e.g. Sheppard & Bate 1980; Hoorn 1993, 1994; Räsänen *et al*. 1995; Gingras *et al*. 2002; Wesselingh *et al*. 2002; Vonhof *et al*. 2003; Hovikoski *et al*. 2005, 2007, 2010; Rebata *et al*. 2006; Linhares *et al*. 2011; for recent compilation of arguments in favour see Boonstra *et al*. 2015). Nonetheless, the evidence is equivocal and permits differing interpretations (Cozzuol 2006; Westaway 2006; Latrubesse *et al*. 2007, 2010; Lundberg *et al*. 2010; Riff *et al*. 2010; Silva-Caminha *et al*. 2010; Gross *et al*. 2011, 2013; for comparable discussions see, e.g. Nicolaidis & Coimbra 2008; Ruskin *et al*. 2011).

Aside from mangrove-related pollen, dinoflagellate cysts, foraminiferal linings and remains of several marine vertebrate clades, body fossils that are specific for marine environments are scarce and restricted to thin intervals (e.g. Linhares *et al*. 2011; Boonstra *et al*. 2015). Remarkably, the highly endemic aquatic invertebrate fauna of the ‘Pebas system’ is strongly dominated by the abundance of pachydontine bivalves and the cytheroid ostracod *Cyprideis*. Otherwise common freshwater and marine taxa are rare and typical marginal marine molluscs (e.g. arcids, oysters, mangrove cerithioidean snails) are absent (e.g. Whatley *et al*. 1998a; Wesselingh 2006, 2007; Gross *et al*. 2013, 2014). Based on the mollusc and ostracod faunas, brackish waters (e.g. Purper 1979; Nuttall 1990; Whatley *et al*. 1998a) or extensive marine transgressions (Sheppard & Bate 1980) were proposed. However, geochemical investigations performed on the shells of these biota consistently indicate freshwater conditions. Elevated salinity (∼5 PSU) is only evident for a few localities (e.g. Vonhof *et al*. 2003; Kaandorp *et al*. 2006; Wesselingh *et al*. 2006; Gross *et al*. 2013).

Wesselingh (2007) considered the ‘Pebas system’ to be a predominantly freshwater environment, in which adaptations to predation pressure and a muddy, poorly oxygenated substrate triggered speciation, as well as habitat dominance by pachydontine bivalves. Gross *et al*. (2013) suggested that it was a locally unstable but on a regional scale long-lived wetland, where euryoecious biology, passive dispersal predispositions and reproduction modes (brood care) favoured the success of the genus *Cyprideis*. Thus, a conclusive, ‘simple’ explanation about the nature of this ‘mega-wetland’ is still pending.

**Figure 1.**
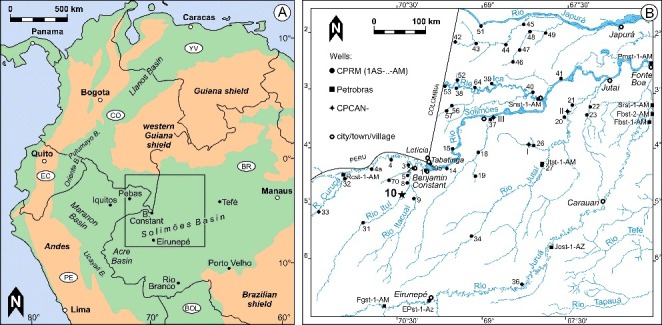
Location of the studied well 1AS-10-AM in western Amazonia. **A,** overview map; **B,** position of exploration wells (after Maia *et al*. 1977); star = herein investigated core; compare Gross *et al*. (2014).

The current study was initiated by the finding of a very small-sized, marine ostracod (*Pellucistoma*) in late middle Miocene sediments of western Brazil (Fig. 1). This posed three central questions: (1) does this record prove the existence of marine incursions, thousands of kilometres away from the next (palaeo-)coastline; (2) which provenance could *Pellucistoma* be from; and (3) what are the potential migration pathways? It is certainly tempting to approve the first question by applying uniformitarian principles, to suppose a Caribbean origin (as suggested for e.g. molluscs; Wesselingh & Ramos 2010) and to relate our record directly to far-reaching marine incursions (e.g. Boonstra *et al*. 2015). However, in view of the conflicting discussions about this topic, we performed an extensive taxonomic, ecological and biogeographical appraisal of *Pellucistoma*. Based on this, we explore possible answers to the above-mentioned questions.

## Geological setting and age

The investigated material originates from a well (1AS-10-AM) drilled ∼62 km south-west of Benjamin Constant in western Brazil (site Sucuriju, close to Rio Ituí; 04 ° 50′ S, 70 ° 22′ W; state of Amazonia; Fig. 1). Based on the available subsurface information (Maia *et al*. 1977; Del' Arco *et al*. 1977), it is located in the Solimões Basin (e.g. Wanderley-Filho *et al*. 2010) and penetrates (except Holocene soils) sediments of the Solimões Formation (for substantial discussions about this formation see Purper 1979; Hoorn 1994; Latrubesse *et al*. 2010; Hoorn *et al*. 2010b).

Well 1AS-10-AM was continuously cored down to 400.25 m. Its lithology consists of alternations of semi-indurated clay and silt. Up to metre-thick, sandy as well as decimetre-thick, lignite intercalations occur subordinately. Recently, Gross *et al*. (2014) studied the microfauna (in particular the ostracod genus *Cyprideis*). For more detailed core descriptions and an illustration of the section, we refer to this work. The herein discussed findings stem from sample AM10/30 (depth = 141.2 m), which is a clayey silt, rich in mollusc remains. According to Gross *et al*. (2014) this sample is biostratigraphically dated to the *Cyprideis obliquosulcata* ostracod zone *sensu* Muñoz-Torres *et al*. (2006), corresponding to a late middle Miocene age (Wesselingh & Ramos 2010).

## Material and methods

Samples (250 g of dried sediment; 40 °C, 24 h) from core 1AS-10-AM were washed through standard sieves (63/125/250/500 µm) using diluted hydrogen peroxide for disintegration (H_2_O_2_:H_2_O = 1:5). Wet sieve residuals were washed with ethanol (70%) before drying (40 °C, 24 h; Gross *et al*. 2014). Residuals ≥125 µm of sample AM10/30, from which the species under discussion originates, were picked out completely for their micropalaeontological content.

**Figure 2.**
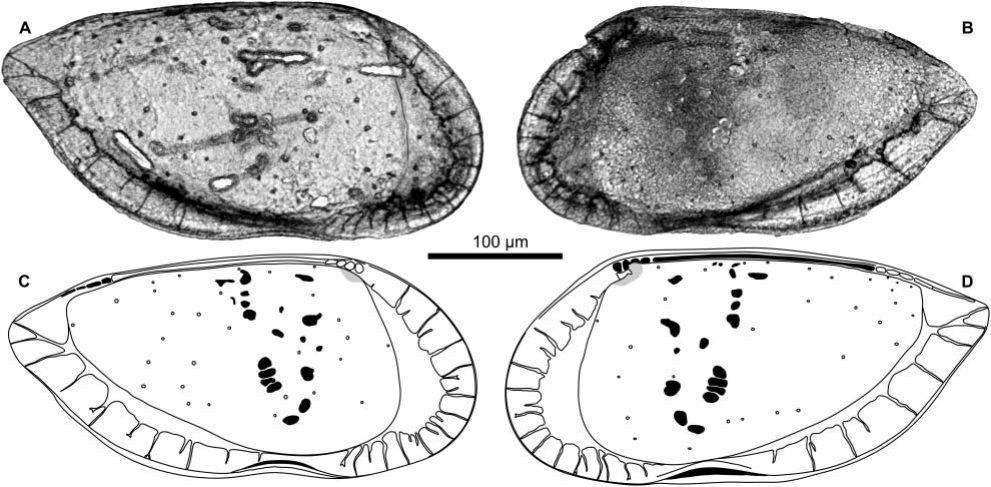
Transmitted light photographs (**A, B,** focus stacked) and schematic drawings (**C, D**) of *Pellucistoma curupira* sp. nov. **A,** MPEG-513-M, left valve, internal view (length = 0.37 mm, height = 0.18 mm); **B,** MPEG-509-M, right valve, internal view of Figure 3F; **C,** left valve, internal view, based on A and Figure 3E; **D,** right valve, internal view, based on B and Figure 3H (compare also Fig. 3F).

Prior to scanning electron microscope imaging (SEM: JEOL JSM-6610LV), the shells were photographed in transmitted light (Leitz Orthoplan microscope, camera: Leica DFC290) and measured (Leica Application Suite V3.6.0). Focus stacked images (Fig. 2A, B) were obtained by combining ∼35 transmitted light photographs per specimen (covered with distilled water) with the software CombineZP.

For stable isotope analyses (δ^18^O, δ^13^C), ostracod valves were additionally washed with distilled water and rinsed in ethanol. From core 1AS-10-AM (sample AM10/30) adults of three species were measured: *Cyprideis machadoi* (Purper, 1979), *Cyprideis multiradiata* (Purper, 1979) and *Cyprideis sulcosigmoidalis* (Purper, 1979) (for taxonomy see Gross *et al*. 2014). The number of specimens required for analyses (∼50 µg) varied between 1 and 3 per measurement. A Thermo-Finnigan Kiel II automated reaction system and a Thermo-Finnigan Delta Plus isotope-ratio mass spectrometer were used to conduct the analyses (University of Graz; standard deviation = 0.1‰ relative to NBS-19; results in per mille relative to the Vienna Pee Dee Belemnite (VPDB) standard).

All specimens are housed in the micropalaeontological collection of the Museu Paraense Emílio Goeldi, Belém (Inv. No. MPEG-503-M to MPEG-514-M).

## Systematic palaeontology

Suprageneric classification follows Horne *et al*. (2002).

Class **Ostracoda** Latreille, 1802

Order **Podocopida** Sars, 1866

Superfamily **Cytheroidea** Baird, 1850

Family **Cytheromatidae** Elofson, 1939

Genus ***Pellucistoma*** Coryell & Fields, 1937

#### Type species

*Pellucistoma howei* Coryell & Fields, 1937.

***Pellucistoma curupira*** sp. nov.

(Figs 2, 3)

**Figure 3.**
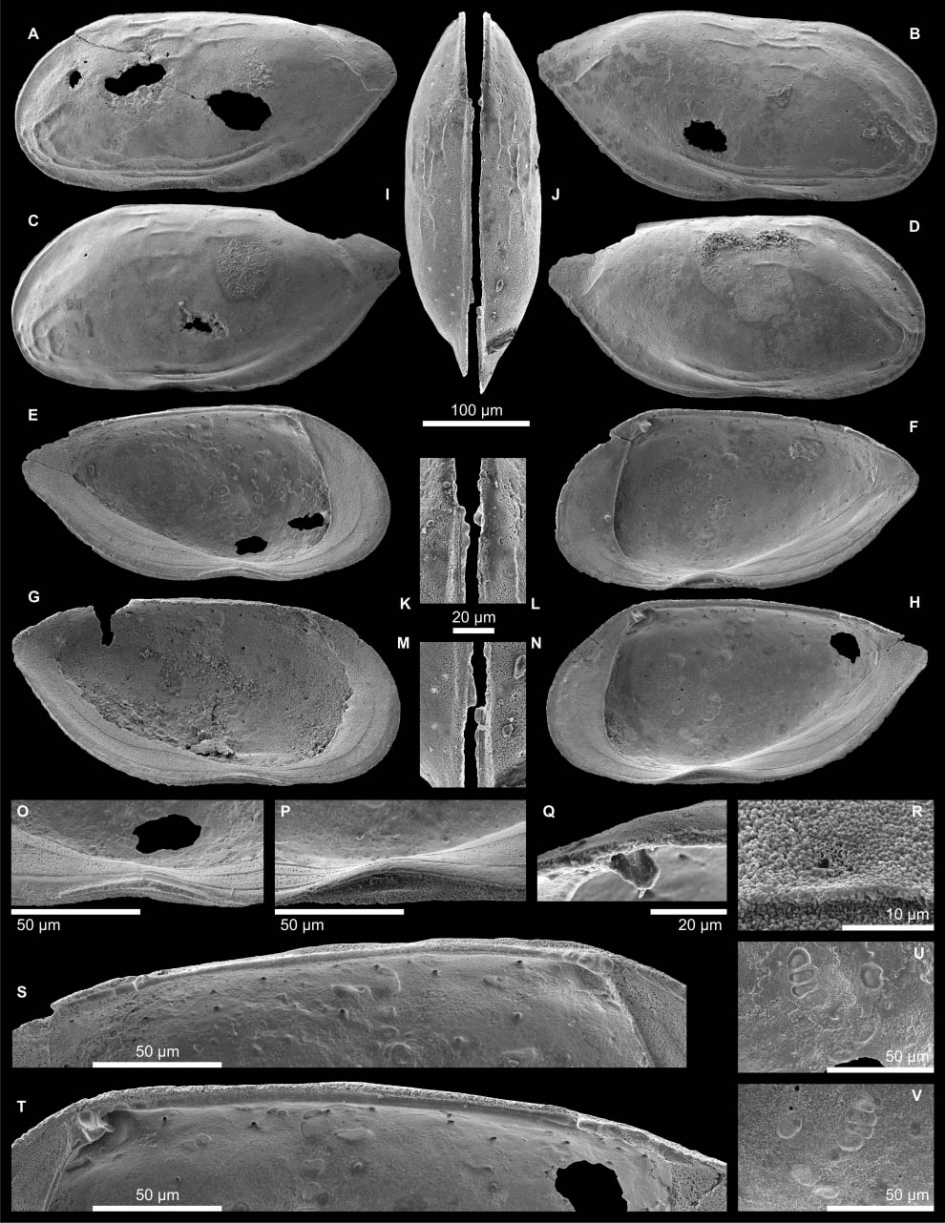
*Pellucistoma curupira* sp. nov. **A,** MPEG-504-M, left valve, external view (length = 0.36 mm, height = 0.17 mm); **B,** MPEG-505-M, right valve, external view (length = 0.38 mm, height = 0.18 mm); **C,** MPEG-506-M, left valve, external view (length = 0.38 mm, height = 0.18 mm); **D,** MPEG-507-M, right valve, external view (length = 0.36 mm, height = 0.18 mm); **E,** MPEG-508-M, left valve, internal view (length = 0.35 mm, height = 0.17 mm); **F,** MPEG-509-M, right valve, internal view (length = 0.34 mm, height = 0.17 mm); **G,** MPEG-510-M, left valve, internal view (length = 0.37 mm, height = 0.18); **H,** holotype MPEG-503-M, right valve, internal view (length = 0.36 mm, height = 0.18 mm); **I,** MPEG-511-M, left valve, dorsal view (length = 0.34 mm, height = 0.17 mm); **J,** MPEG-512-M, right valve, dorsal view (length = 0.36 mm, height = 0.17 mm); **K,** anterior hinge element of I; **L**, anterior hinge element of J; **M,** posterior hinge element of I; **N,** posterior hinge element of J; **O,** ventral concavity of E; **P,** ventral concavity of H; **Q,** anti-slip tooth of J (oblique dorsal view); **R,** normal pore, sieve-type of B; **S,** hinge of E; **T,** hinge of H; **U,** central muscle scars of E; **V,** central muscle scars of H.

#### Holotype

MPEG-503-M, right valve (Fig. 3H, P, T, V).

#### Paratypes

MPEG-504-M to MPEG-513-M (Figs 2A, B, 3A–G, I–O, Q–S, U).

#### Additional material

MPEG-514-M, 26 adult specimens from sample AM10/30.

#### Diagnosis

A very small-sized, extremely thin-shelled species of *Pellucistoma* with subrhomboidal shape, ornamented with wrinkle-like ridges forming anteroventrally a weak reticulum and a unique combination of hinge structures.

#### Derivation of name

‘*Curupira*’, the name of a mythic dwarf of Brazilian legends with backward turned feet, which should confuse pursuers; used as a noun in apposition; in reference to the small size of the species and its baffling discovery.

#### Type locality

Borehole 1AS-10-AM at Sucuriju close to Rio Ituí (04 ° 50′ S, 70 ° 22′ W, ∼62 km south-west of Benjamin Constant; municipality Atalaia do Norte, state of Amazonia, Brazil; Fig. 1).

#### Type horizon

Sample AM10/30 ( = depth: 141.2 m, altitude: –56.2 m; Gross *et al*. 2014).

#### Description

#### Shape

Subrhomboidal in lateral view; anterior margin moderately infracurvate, dorsal margin almost straight and subhorizontal, ventral margin with slight concavity below the mandibular scars, posterior margin with a blunted subdorsal caudal process; valves anterodorsally, posteroventrally and posteriorly laterally flattened; lens-shaped in dorsal view with beaked posterior end.

##### Ornamentation

The very thin-shelled valves are basically smooth except for shallow, wrinkle-like ridges along the free valve margin as well as in the centro- and ventrodorsal area, forming a weak reticulum anteroventrally; at the caudal process an additional, oblique ridge is always present.

##### Inner lamella

Anterior and posterior wide; anterior vestibulum large, posterior one narrow-elongated, extending up to the caudal process; several inner lists developed; selvage subperipheral, inconspicuous anteriorly, forming at the ventral concavity a bulge, which fits into a groove of the other valve (groove more prominent in right valves).

##### Marginal pore canals

Widened at their base, leading to an irregular line of concrescence; occasionally bifurcated, some branches developed as false pore canals.

##### Hinge

Right valve – anterior element with four roundish sockets (the most anterior being the largest, the most posterior one is barely developed) and a spatulate anti-slip tooth below; median element consists of a smooth groove, which is deepened at its posterior end; posterior hinge element with three elongated teeth, succeeded by a fourth, indistinct tooth, which merges backwards into a thin expansion of the posterior margin; left valve – anterior element with a trilobate tooth and a further weak tooth postjacent; median element consisting of a smooth bar, which forms an elongated tooth-like structure at its posterior end; posterior element with three, elongated, shallow sockets followed by a fourth, indefinite, elongated socket fading out towards the posterior end.

##### Normal pores

Widely scattered, very small (∼3–5 µm in diameter); sieve-type.

##### Central muscle scars

A row of four, slightly posteriorly inclined adductor scars; two oval mandibular scars, one irregularly ovate frontal scar; numerous dorsal muscle scars; well above the row of adductor scars a row of four dorsal scars is developed, probably corresponding to the ‘lucid spot’ (Morkhoven 1963; Sandberg 1969), which, however, is not a discrete, single spot here.

##### Eye-spot

Slight eye-spot developed.

##### Sexual dimorphism

Unclear; a few specimens (e.g. Fig. 3B) are slightly larger and display a somewhat higher posterior valve proportion, which could be related to sexual dimorphism.

##### Dimensions

Right valve (number of measured specimens = 9): length = 0.34–0.38 (mean = 0.36) mm, height = 0.17–0.18 (mean = 0.18) mm; left valve (number of measured specimens = 7): length = 0.34–0.38 (mean = 0.36) mm, height = 0.17–0.18 (mean = 0.17) mm.

#### Remarks

##### Generic classification

The current species imitates several genera of different families by its subrhomboidal outline and almost smooth shells.

Amongst the Bythocytheridae Sars, 1866, some species of *Bythocythere* Sars, 1866 and *Pseudocythere* Sars, 1866 superficially resemble *Pellucistoma curupira* sp. nov. However, both genera differ explicitly due to the development of five adductor scars, and, less clearly, in their hinges (*Pseudocythere*: adont; *Bythocythere*: adont, lophodont or merodont; e.g. Morkhoven 1963; Athersuch *et al*. 1989; Stepanova 2006; Sciuto 2009).

The loxoconchid genera *Palmoconcha* Swain & Gilby, 1974 ( = syn. *Lindisfarnia* Horne & Kilenyi, 1981; Horne & Whatley 1985; Athersuch *et al*. 1989), *Elofsonia* Wagner, 1957, *Pseudoconcha* Witte, 1993 and, especially, *Phlyctocythere* Keij, 1958, are similar to some degree.

However, *Palmoconcha* is distinguished by its gongylodont hinge (right valve: anterior socket–tooth–socket sequence; median smooth furrow; posterior tooth–socket–tooth sequence; Swain & Gilby 1974; Horne & Kilenyi 1981; Athersuch & Horne 1984; Horne & Whatley 1985; Athersuch *et al*. 1989) and to a minor degree by its less prominent caudal process, strong fulcral point and Y-shaped frontal scar.

*Pseudoconcha* has a bipartite hinge (right valve: anterior element formed by a strong bar, with a groove below; posterior half with groove and bar below), a less developed caudal process, a well-punctate surface and a narrower inner lamella (Witte 1993; Sarr *et al*. 2008).

Although some variability in details of the hinge, muscle scar patterns and pore canals seem to be present in *Elofsonia* (Aiello & Szczechura 2002), this genus differs by its less prominent, less pointed caudal process (except *Elofsonia* sp. in Keyser & Schöning 2000) and its more simple hinge (right valve: dorsally crenulated anterior socket; smooth median groove; weak posterior tooth; Whittaker 1973; Athersuch & Horne 1984; Athersuch *et al*. 1989).

Originally, *Phlyctocythere* was characterized by its inflated, almost spherical carapaces with a peripherally compressed zone and an obtuse, subdorsal caudal process. Its surface is smooth, lacks eye-spots and the valves are very thin-shelled. The hinge is adont (right valve: curved, smooth bar), marginal pore canals are simple, and one frontal muscle scar is developed (Keij 1958; compare also Morkhoven 1963). Subsequently, several species were included in Phlyctocythere, which blur the prime generic diagnosis. For example: (1) outline: *Phlyctocythere hamanensis* Ikeya & Hanai, 1982 (more elongated, less arched dorsal margin), *Phlyctocythere japonica* Ishizaki, 1981 (subovate), *Phlyctocythere recta* Bold, 1988 (straight dorsal margin), *Phlyctocythere sicula* Sciuto & Pugliese, 2013 (more elongated) and *Phlyctocythere stricta* Bold, 1988 (straight dorsal margin); (2) ornament: *Phlyctocythere curva* Bold, 1988, *Phlyctocythere retifera* Bonaduce, Masoli & Pugliese, 1978, *P. sicula* and *P. stricta* display a faint reticulation and/or longitudinal ridges/wrinkles; (3) *Phlyctocythere curva*, *P. recta*, *P. stricta* and probably *P. fennerae* Mostafawi, 1992 have a slight eye-spot; (4) for *P. fennerae*, *P. japonica* and *P. retifera* few (anteroventrally) branched marginal pore canals are described and these structures are often observed to be bifurcated in *P. curva*; (5) for *P. hamanensis* normal pores are ‘presumably’ of sieve-type; (6) in *P. hamanensis* two frontal scars and one elongated mandibular scar are mentioned; in the illustration of *P. retifera* a double frontal scar is indicated; in *P. sicula* the adductor scars are very elongated; and (7) hinge: in *Phlyctocythere caudata* Hartmann, 1979, *P. curva*, *Phlyctocythere pellucida* (Müller, 1894), *P. retifera* and *P. sicula* right valves display a smooth median groove (crenulated in *P. curva*) and (two) terminal sockets (in *P. curva*: teeth); *P. hamanensis* has a reduced gongylodont hinge. (Note: *Phlyctocythere hartmanni* Omatsola, 1970 is attributed to *Elofsonia* (Athersuch & Horne 1984; Schornikov 2011) or to *Pseudoconcha* (Witte 1993). *Phlyctocythere pellucida* is discussed as belonging to *Loxocauda* Schornikov, 1969 (Athersuch & Horne 1984; Schornikov 2011)). Consequently, *Phlyctocythere* is either quite variable or has turned into a collective genus due to inclusion of profuse species. An in-depth revision is obviously needed but is beyond the scope of the present work. In particular, *P. retifera* from the Red Sea is similar but it is more ovate (but note sexual dimorphism displayed in Mostafawi (1992) for *P. fennerae*), it diverges in details of the hinge (as far as reproducible) and has two frontal scars. However, by following the original diagnosis of *Phlyctocythere* (Keij 1958; Schornikov 2011) especially its outline (much more arched dorsal margin), its adont, right-bar hinge and simple marginal pore canals are considered herein to exclude an assignment of the current specimens to that genus.

Some authors (Bold 1950, 1958; Benson *et al*. 1961; Morkhoven 1963) have discussed a possible synonymy of *Javanella* Kingma, 1948 with *Pellucistoma* Coryell & Fields, 1937 (see also Gou & Chen 1988; Howe & McKenzie 1989; Ayress 1996). Lately, Bergue & Coimbra (2007) revised *Javanella*, revalidated it and reassigned it into the family Cytheridae Baird, 1850. According to this work only two species are left in *Javanella*, which clearly differ in outline (more elongated; caudal process below the middle of valves height) and in details of the terminal hinge elements from the present material.

Amongst the Cytheromatidae Elofson, 1939, the genus *Paracytheroma* Juday, 1907 is closely related to *Pellucistoma*. Nevertheless, *Paracytheroma* can be differentiated from the latter by lacking strong terminal anti-slip hinge elements, the absence of a caudal process and – to a minor degree – by missing a complex marginal zone with branched marginal canals (Hartmann 1978; Ayress 1990; compare also Sandberg 1969; Keyser 1976; Garbett & Maddocks 1979). Based on those features, our specimens do not belong to *Paracytheroma* but fit best with *Pellucistoma* as originally defined by Coryell & Fields (1937; for genus definition compare also Edwards 1944; Morkhoven 1963; Sanguinetti 1979). A few, minor differences concern the valves’ hinge and surface ornament.

For the hinge of the left valve an “anterior long blade-like triangular tooth” is indicated (Coryell & Fields 1937, p. 17), being trilobate in the present specimens. The median element is formed by a “serrated bar”, which “terminates at the posterior cardinal angle” (Coryell & Fields 1937, p. 17). Here, that bar is smooth – at least as preserved. As far as described or perceptible on the provided figures, a smooth median element occurs in *Pellucistoma scrippsi* Benson, 1959 and *Pellucistoma bensoni* McKenzie & Swain, 1967 (Benson & Kaesler 1963; McKenzie & Swain 1967; Swain & Gilby 1967, 1974). For the type species, *P. howei*, the drawings of Bold (1967) and Teeter (1975) do not show such a crenulation. The crenulation of the median hinge element is in some species of *Pellucistoma* probably very delicate or indeed not developed.

A posterior hinge element, consisting of four elongated sockets/teeth as in our examples, has not been mentioned for *Pellucistoma* so far. However, based on the dorsal view of a left valve in Coryell & Fields (1937), behind the thickened, tooth-like terminal end of the bar, a shallow groove may be present which might analogously receive tiny teeth of the right valves. Moreover, the illustration of *P. scrippsi* in Swain & Gilby (1967) implies the presence of small posterior teeth in the right valve (note that this feature is not indicated in e.g. Benson 1959; Benson & Kaesler 1963; McKenzie & Swain 1967; Swain 1967). The description and illustration of the hinge structure of *Pellucistoma spurium* Bold, 1963 (p. 406: “In the left valve the selvage curves around the sockets and forms the upper border of the groove”) also hints at the presence of a posterior element. Thus, the subtle posterior sockets/teeth, clearly visible in our species (under the SEM), seem to be present in other *Pellucistoma* species equally. Garbett & Maddocks (1979, p. 871) carefully described a similar posterior hinge structure for *Paracytheroma stephensoni* (Puri, 1954) of which *Pellucistoma atkinsi* Hall, 1965 is a synonym (Keyser 1976): “[a] posterior tooth formed by the expanded end of the posterior margin.” That resemblance mirrors the close relation between *Pellucistoma* and *Paracytheroma* again (see above).

The surface of *Pellucistoma* is described as “finely perforated” (Coryell & Fields 1937, p. 17) and “smooth or finely punctate” (Morkhoven 1963, p. 436). Here, the valves are basically smooth, but display a weak reticulate pattern anteroventrally, some wrinkle-like ridges dorsocentrally and ventrocentrally, as well as a characteristic, oblique, light ridge on the caudal process. Shallow, posterocentral and posteroventral ridges, which converge towards the caudal process, can be seen on *P. magniventra* in Garbett & Maddocks (1979). True eye-spots have not been included in the genus definition so far. However, a slight eye-spot – like in *P. curupira* sp. nov. – is recognized in *P. scrippsi* (Swain 1967; Swain & Gilby 1974).

##### Comparison with other *Pellucistoma* species

To our knowledge, 15 *Pellucistoma* species have been formally described so far (e.g. Kempf 1986, 1995, 2008; Brandão 2015).

The type species, *Pellucistoma howei* Coryell & Fields, 1937 (first record: Panama, latest middle–early late Miocene), is quite similar but differs by: its more ovate outline; its more projecting posteroventral margin; a more acuminate caudal process; a narrower anterior vestibulum (which seems to be almost restricted to the lower half of valve height); the ventral snap-mechanism is less developed; and its larger size (holotype: length/height = 0.48/0.27 mm; Coryell & Fields 1937; Bold 1967; see also e.g. Teeter 1975; Bold 1988). For differences in hinge and ornamentation see above.

*Pellucistoma magniventra* Edwards, 1944 (first record: North Carolina, Pliocene) is distinguished by (largely based on the redescription of Garbett & Maddocks 1979): its more arched, upwards rising dorsal margin and its strongly projecting posteroventral margin, respectively (if not aligned to the base line = reversal points in front and backwards of the ventral concavity); its much more infracurvate anterior margin; its more pointed and acuminate caudal process; its ornament (see above); its anterior vestibulum, which is largely restricted to the anteroventral area (for variability see Garbett & Maddocks 1979); details of the hinge (crenulated median element; simple anterior and posterior sockets/teeth; lack of posterior hinge elements); and its larger size (holotype: length/height = 0.62/0.33 mm; Edwards 1944; Garbett & Maddocks 1979; see also e.g. Puri 1960; Bold 1963; Benson & Coleman 1963; Hall 1965; Morales 1966; Grossman 1967; Sandberg 1969; Cronin 1979; Krutak 1982; King Lyon 1990).

*Pellucistoma scrippsi* Benson, 1959 (first record: Baja California, Recent) has more convex dorsal and ventral margins and a less oblique anterior margin as well as a smooth surface. The posterior vestibulum is almost absent (but see Swain & Gilby 1967); the hinge is slightly different (simple anterior socket (right valve) and tooth (left valve); posterior teeth are lacking (except illustration of Swain & Gilby 1967)); marginal pore canals are missing on the apex of the caudal process; and it is larger (holotype: length/height = 0.69/0.33 mm; Benson 1959; Benson & Kaesler 1963; McKenzie & Swain 1967; Swain 1967; Swain & Gilby 1967, 1974).

*Pellucistoma bensoni* McKenzie & Swain, 1967 (first record: Baja California, Recent) differs in its strongly arched dorsal margin resulting in a subtriangular outline and a posterocentrally located caudal process, its smooth surface, its almost absent posterior vestibulum; and it is larger (holotype: length/height = 0.44/0.25 mm).

*Pellucistoma spurium* Bold, 1963 (first record: Trinidad, late Miocene) has a more convex dorsal margin; a more accentuated caudal process; and a hinge with a simple knob-like anterior tooth (left valve) and minutely crenulated median elements. The anterior vestibulum is restricted to the anterocentral area, the posterior vestibulum is only developed at the caudal process; and it is larger (holotype: length/height = 0.49/0.25 mm; compare also *Pellucistoma*? *spurium* of Bold (1988)).

*Pellucistoma santafesinensis* Zabert, 1978 (first record: Argentina, middle–late Miocene; correct spelling according to Kempf (2008): *P. santafesinense*; note: in the following we refer to correct spellings of species names but retain the original spellings in this work) is similar to *Pellucistoma gibosa* Sanguinetti, 1979 (see below) and possibly both are synonyms. However, it differs significantly in outline (subtriangular-elongate in lateral view; ventromedian long, pointed caudal process) and hinge structures from the current material. Size of holotype: length/height = 0.53/0.26 mm.

*Pellucistoma gibosa* Sanguinetti, 1979 (first record: southern Brazil, late Miocene; correct spelling according to Kempf (1986): *P. gibosum*) has an extremely humped (right valve) dorsal margin and an acuminate, long caudal process well below the half valves’ height. It is smooth; it has a hinge with a simple, strong anterior tooth (left valve) and a slightly serrated bar; the anterior vestibulum is restricted to lower half of valves height; and it is larger (holotype: length/height = 0.51/0.23 mm).

*Pellucistoma elongata* Whatley *et al*., 1997a (first record: Argentina, Recent; correct spelling according to Kempf (2008): *P. elongatum*) differs by: its more convex dorsal and posteroventral margin; more acuminate and more ventrally located caudal process; the inner lamella curves inwards posteroventrally; it is smooth (lacks eye-spots); and it is larger (holotype: length/height = 0.52/0.24 mm; Whatley *et al*. 1997a).

##### Further species of questionable generic classification

Bold (1950) considered his Miocene Venezuelan species *Pellucistoma kendengensis* (Kingma) to be synonymous with *Javanella kendengensis* Kingma, 1948 (Pliocene, Java). Later, Bold (1972a) included *P. kendengensis* of Bold (1950) in his new species *Pellucistoma*? *kingmai* Bold, 1972a and assumed *J. kendengensis* of Kingma (1948) not to be a synonym of *P*.? *kingmai*. Bergue & Coimbra (2007) re-examined the type material of Bold (1950), excluded it from *Javanella* but left the generic status of *P. kendengensis* (according to Bold 1972a: *P*.? *kingmai*) open. Although there are some features perceptible that are unlike *Pellucistoma* (anterior margin almost equicurvate (cf. Bold 1950, p. 86: “obliquely rounded”); subventral caudal process; quite heavily punctate surface (as shown in Bergue & Coimbra 2007), as well as short and straight marginal pore canals, it cannot be definitively excluded from *Pellucistoma*.

Due to their simple marginal pore canals, the species *Pellucistoma*? sp. (Bold 1958, 1972a), *Pellucistoma*? *compactum* Bold, 1972a and *Pellucistoma*? *kingmai* Bold, 1972a, that are questionably attributed to *Pellucistoma*, differ in outline, hinge structure and development of the inner lamella. Only *Pellucistoma* sp. in Bold (1970, 1972b, 1988) is rather similar in its shape to the current species. Nevertheless, it has a smooth surface and is larger (length/height = 0.48/0.25 mm). Unfortunately, neither the hinge nor inner characters are accessible, which does not enable further comparisons.

*Pellucistoma tumida* Puri, 1954 (correct spelling according to Kempf (1986): *P. tumidum*) from the Pliocene of Florida is poorly described and a re-examination of the type material already failed (Bold 1988). Although its outline is comparable with the present individuals (except the equicurvate anterior margin), without additional traits (see also Bold 1963; Hulings 1967) a generic assignment or a species-specific identification is unfeasible. Bold (1988) discussed possible congruence with his *Phlyctocythere* sp. 2, which again demonstrates the superficial similarity of that loxoconchid genus with *Pellucistoma* (see above).

*Pellucistoma atkinsi* Hall, 1965 (see above) is a junior synonym of *Paracytheroma stephensoni* (Puri, 1954) (Keyser 1976; Garbett & Maddocks 1979).

*Pellucistoma ovaliphylla* Hu, 1981 from the Plio-/Pleistocene of southern Taiwan has been recognized by Hu (1984) to belong to *Paradoxostoma* Fischer, 1855. Two further Taiwanese species, *Pellucistoma magnolioidea* Hu & Tao, 2008 and *Pellucistoma chushunshui* Hu & Tao, 2008, differ notably in outline, especially due to the almost lacking caudal process, from both the current as well as from other *Pellucistoma* species (important internal characters are not accessible because of missing illustrations and descriptions). Most likely, these species belong to another genus (*Paracytheroma*?) but this claim needs additional investigations.

Ayress (1990, 1996) described *Pellucistoma coombsi* Ayress, 1990, *Pellucistoma fordycei* Ayress, 1990 and *Pellucistoma punctata* Ayress, 1996 (correct spelling according to Kempf (2008): *P. punctatum*) from New Zealand and the Tasman and Coral Seas. These species diverge significantly from the present species and the genus *Pellucistoma* in general (outline: much more elongated-rectangular; hinge structures: e.g. *P. coombsi* and *P. punctata* have a right-bar hinge; inner lamella: much wider; ornament: *P. punctata*), which shed doubt on their generic allocation. However, those species are not comparable with the material described herein.

The illustration and description of *Pellucistoma* sp. from Henderson Island (Pitcairn Islands, S. Pacific; Recent; Whatley & Roberts 1995; Whatley *et al*. 2004) do not offer enough details (e.g. hinge) for an assured generic attribution. Its laterally inflated valves are rather unlike those of *Pellucistoma*.

Faugères *et al*. (1984) mentioned *Pellucistoma* from the Ghubbet el Kharab (Djibouti; Holocene) but provided no figure. Presumably, this material belongs to another genus (*Phlyctocythere*?).

Whatley *et al*. (1997b) recorded – without figure or description – *Pellucistoma* sp. 1 from the southern Strait of Magellan (Chile; Recent). Due to extremely low water temperatures at the sampling sites, this record is ecologically very unlikely for *Pellucistoma* (see below). Thus, we do not consider it subsequently.

The Late Cretaceous *Pennyella foveolata* Majoran & Widmark, 1998 from the Maud Rise (Southern Ocean, off Antarctica), listed under *Pellucistoma* in the ‘World Ostracoda Database’ (Brandão *et al*. 2015), actually belongs to the former genus (Yasuhara *et al*. 2013).

To conclude, all the above compared species can be clearly differentiated from *Pellucistoma curupira* sp. nov. and are noticeably larger. Most similar are *P. howei* from the Miocene of Panama and *Pellucistoma* sp. of Bold (1970, 1972b, 1988; Miocene: Antilles and Panama), however, the latter is little known and a closer examination is not possible. Most likely, the Australasian and Taiwanese species (Ayress 1990, 1996; Hu & Tao 2008) do not belong to *Pellucistoma*. The records from the Pitcairn Islands and southern Chile (Whatley *et al*. 1997b, 2004) need additional affirmation. Currently, we assume that the genus *Pellucistoma* is confined to the Americas.

## Results

### Stable isotope (δ^18^O and δ^13^C) analyses

**Figure 4.**
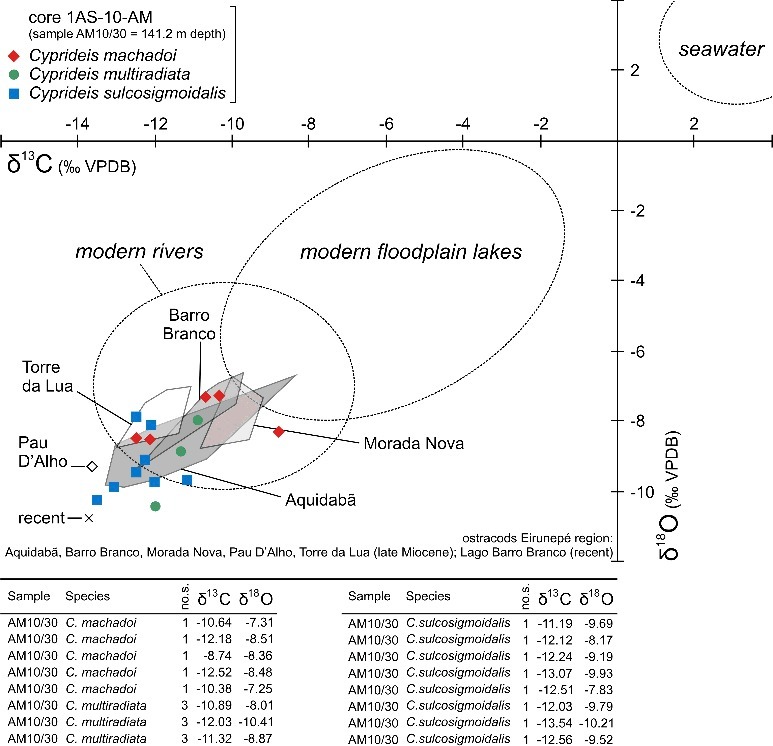
δ^18^O and δ^13^C isotopic ratios of *Cyprideis* species associated with *Pellucistoma curupira* sp. nov. Abbreviation: no.s., number of shells used for analysis. Grey shaded polygons display the range of results obtained from fossil and Recent ostracods from the Eirunepé region (Gross *et al*. 2013). (Note: the indicated range for modern rivers and floodplain lakes is based on aragonitic mollusc shells (Wesselingh *et al*. 2006), which give somewhat heavier values for the same environmental parameters compared to ostracod calcite (Grossman & Ku 1986)).

Due to the minute size of *Pellucistoma curupira* sp. nov., geochemical analyses were performed on three *Cyprideis* species, co-occurring in the same sample (AM10/30). All measurements provided very light values with a range for δ^18^O from –7.25 to –10.41‰ and for δ^13^C from –8.74 to –13.54‰ (Fig. 4).

### Spatiotemporal distribution and autecology of hitherto known *Pellucistoma* species

**Figure 5.**
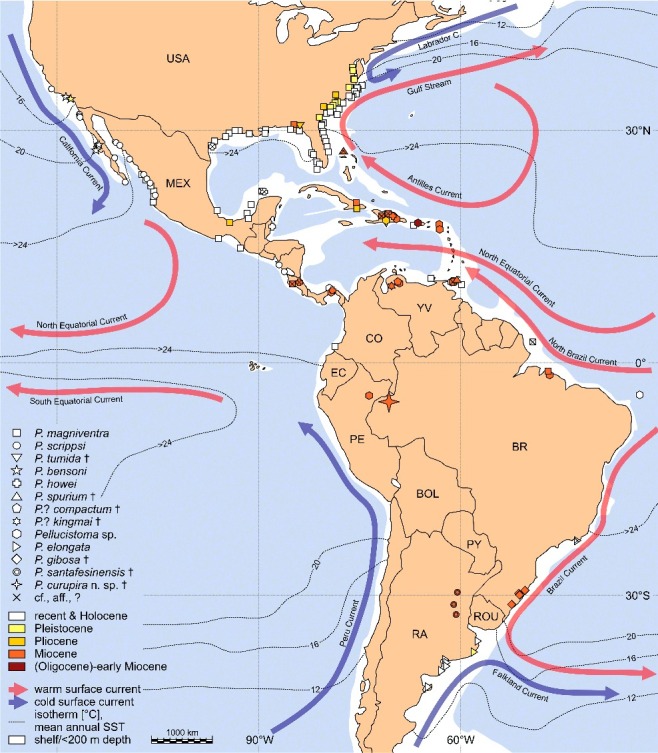
Fossil and Recent records of *Pellucistoma* species (mean annual sea surface temperature (SST) based on NASA data (http://svs.gsfc.nasa.gov/index.html; accessed 18 September 2014); for details see Supplemental Material 1 and 2; species only known from the fossil record marked with †.

We evaluated all published *Pellucistoma* records (fossil and Recent) known to us (Fig. 5; for references and details see Supplemental Material 1 and 2). This review might be incomplete due to overlooked literature and, probably more importantly, because of sampling biases. Partially poor stratigraphical assessment of sampling sites and the inclusion of reports without illustrations may additionally blur our results. Nevertheless, the dataset reveals some significant biogeographical and ecological patterns, which further enable a discussion of the erratic occurrence of *P. curupira* sp. nov. in the Miocene of western Amazonia.

Amongst extant *Pellucistoma* species, *P. magniventra* has a continuous Holocene record along the east coast of the USA (Maryland (∼38 ° N) to southern Florida) and in the northern and southern Gulf of Mexico (Fig. 5). This is the ‘core area’ of *P. magniventra* today. Further scattered indications come from the Pacific coast of Mexico (Sinaloa, Nayarit, Gulf of Tehuantepec) and Colombia (Bahía de Tumaco), from Puerto Rico, Venezuela and Trinidad and the Brazilian equatorial shelf (∼2 ° S; the latter as *P*. ex gr. *magniventra*). To summarize the ecological data (Supplemental Material 1), *P. magniventra* prefers shallow marine (inner sublittoral, ∼10–20 m water depth), subtropical–tropical (minimal water temperature of coldest month (T_min_) ∼10 °C; average annual water temperature (T_av_) >20 °C), oxygenated, euhaline waters (∼30–40 PSU; e.g. Valentine 1971; King Lyon 1990; Fig. 5). Fossil occurrences (late Miocene–Pleistocene) of *P. magniventra* match with the ‘core area’ as outlined above. Additionally, it is reported from Cuba (middle Miocene–Pliocene), Trinidad (early Miocene–Pliocene) and northern Brazil (early Miocene). *Pellucistoma* aff. *magniventra* is noted from Costa Rica (late Miocene) and Hispaniola (middle Miocene–early Pliocene). Palaeoecological data for these fossil occurrences coincide with the ecology of Recent *P. magniventra*.

*Pellucistoma scrippsi*, up to now only with Recent and sub-Recent records, is restricted to the Pacific coast of North and Meso America (∼Santa Barbara/USA (∼34 ° N) to San Juan del Sur/Nicaragua (∼11 ° N)). The rare Recent and Pleistocene findings of *P. bensoni* plot within the distribution area of *P. scrippsi*. For *P. scrippsi* and *P. bensoni* the following autecological data can be summarized: shallow marine (inner sublittoral, ∼10 m water depth), warm temperate–tropical (T_min_ ∼12 °C, T_av_ >15 °C), euhaline.

*Pellucistoma howei*, although principally a fossil species (Panama and Dominican Republic; middle–late Miocene), is recorded from the Holocene of Belize as well as with uncertainty (*P*. cf. *howei* or *P*. aff. *howei*) from the Gulf of Mexico (Recent) and the Miocene of Costa Rica. Ecological data characterize it as a shallow marine (sublittoral, ∼5–50 m water depth), tropical–subtropical, euhaline species.

Except for a few further Recent *Pellucistoma* sp. records (Panama, Trinidad, Rocas Atoll/Brazil), *P. elongata* remains the last extant species to be discussed. It is confined to the Argentinean coast from about the Isla de Los Pájaros (∼42 ° S) towards the north (∼36 ° S) to the Río de la Plata estuary (with one Pleistocene report: Mar Chiquita). Further in the north, *P*. cf. *elongata* is noted off Cabo Frio (Rio de Janeiro, ∼23 ° S; subrecent). Based on the available information, *P. elongata* is a shallow marine (littoral–inner sublittoral; littoral rock pools to ∼14 m water depth), euhaline species. It is reported from shallower settings than the species treated above, however, at least T_min_ can be assumed to be comparable to the occurrences of other species (e.g. Mar del Plata: T_min_ ∼10 °C, T_av_ ∼15 °C; www.seatemperature.org, accessed 12 January 2015).

*Pellucistoma tumida* (Florida), *P. spurium* (Trinidad, ?Bahamas), *P*.? *compactum* (Venezuela), *P*.? *kingmai* (Venezuela), *P*. *gibosa* (Brazil) and *P*. *santafesinensis* (Argentina) are exclusively fossil taxa. As far as it is known, these species, as well as further fossil *Pellucistoma* records left in open nomenclature, lived in shallow marine, subtropical–tropical, euhaline habitats.

In summary, *Pellucistoma* is unquestionably a marine (sublittoral, euhaline) taxon of subtropical–tropical, oxygenated waters with a seasonally lower temperature limit of about 10 °C. All fossil records point in the same direction. So far, there is no evidence for a different autecology in the geological past.

## Discussion

### Constraints of dispersal and potential dispersal modes of *Pellucistoma*

Dispersal capacity of organisms depends on various biotic and abiotic factors (e.g. autecology, reproduction mode, predation, competition, active/passive dispersal capacity, medium of transport). In the case of *Pellucistoma*, water temperature, depth and salinity appear to be the most important physico-chemical parameters (e.g. Valentine 1971, 1976; Bold 1974; Cronin 1979; Cronin & Dowsett 1990). Little is known about its reproduction; however, due to the proof of female and male individuals in some species, sexual reproduction can be assumed. As female carapaces lack an apparent brood pouch (for storage of eggs and/or early instars), brood care seems unlikely. Planktonic larval stages – like in all marine podocopid ostracods – are missing (Titterton & Whatley 1988). Presumably, *Pellucistoma* belongs to the marine meiobenthos. Synecological information (e.g. predation, competition) is not available for this genus.

By considering the small size of *Pellucistoma* and the lack of planktonic stages, active dispersal can be expected to be very slow (Sandberg 1964). Land bridges (e.g. Panamian isthmus), deep-water areas (e.g. Cayman Trench), cold waters/ocean currents (e.g. Peru Current) as well as massive river discharge (e.g. Amazon River; lowering of salinity, instability of the seabed) will be effective dispersal barriers for *Pellucistoma* (compare Cronin 1987; Coimbra *et al*. 1999; Iturralde-Vinent & MacPhee 1999).

However, passive dispersal by animals (e.g. birds, fishes), wind, water currents (fluvial and marine) or, in modern times, by man, are frequently quoted to affect ostracod migrations (e.g. Mesquita-Joanes *et al*. 2012).

Birds can transport ostracods (adults, juveniles, eggs) on e.g. their feet or feathers, preferably encased in sediment. In addition, intestinal transport (and survival) has been successfully demonstrated (e.g. Löffler 1964; Frisch *et al*. 2007; Brochet *et al*. 2010). Although bird-mediated transport is conceivable for *Pellucistoma*, three counter-arguments should be mentioned (Teeter 1973): (1) species of this genus (except *P. elongata*) prefer sublittoral settings, which makes their adhesion to shorebirds/waterfowls difficult; (2) *Pellucistoma* is rather small and thin-shelled (*P. curupira* sp. nov. is very small and very thin-shelled) and thus prone to digestion (also by fishes); and (3) torpid stages, desiccation-resistant eggs (as in freshwater Cypridoidea; e.g. Horne 1993; Rossi *et al*. 2011) and brood care (as e.g. in the cytheroid *Cyprideis*; Sandberg 1964; Bold 1976) facilitate aerial dispersal via birds, but the presence of such features is implausible for *Pellucistoma*. Comparable arguments seem to rule out transport by wind.

Aquatic displacement by rivers (downstream) and by tidal currents (up- and downstream) appears unlikely for the euhaline, sublittoral *Pellucistoma* (Barker 1963). Conversely, ocean currents are regarded as a prominent means of passive ostracod transport (e.g. Titterton & Whatley 1988). Especially, drifting aquatic plants/algae – inclusively adhering sediments – significantly contribute to ostracod dispersal (e.g. Teeter 1973; Cronin 1987).

Human induced dispersal by ships (e.g. in ballast water or on fouling of the hull) has been inferred to influence modern ostracod distribution (Teeter 1973; Witte & Harten 1991). Although the evidence is quite poor, we would refer to the possibility of phoretic dispersal via larger aquatic animals. For instance, manatees (Sirenia: Trichechidae) carry diverse epibionts on their skin (e.g. barnacles, copepods, algae and ostracods; Hartman 1979; Suárez-Morales *et al*. 2010; Marsh *et al*. 2011). Possibly, such animals act – like vessels – as means of dispersal for shallow marine ostracods. In this case, the sublittoral lifestyle of *Pellucistoma*, its susceptibility to intestinal digestion and decease due to desiccation, will be no constraint, as in bird transport. In addition, stream, tidal and ocean currents can be surmounted.

In conclusion, due to the lack of specific, dispersal-favouring traits and its autecology, the colonization of new habitats is subject to more restrictions compared to, for example, the euryoecious, brood care practicing *Cyprideis*.

### Origin and dispersal of *Pellucistoma*

**Figure 6.**
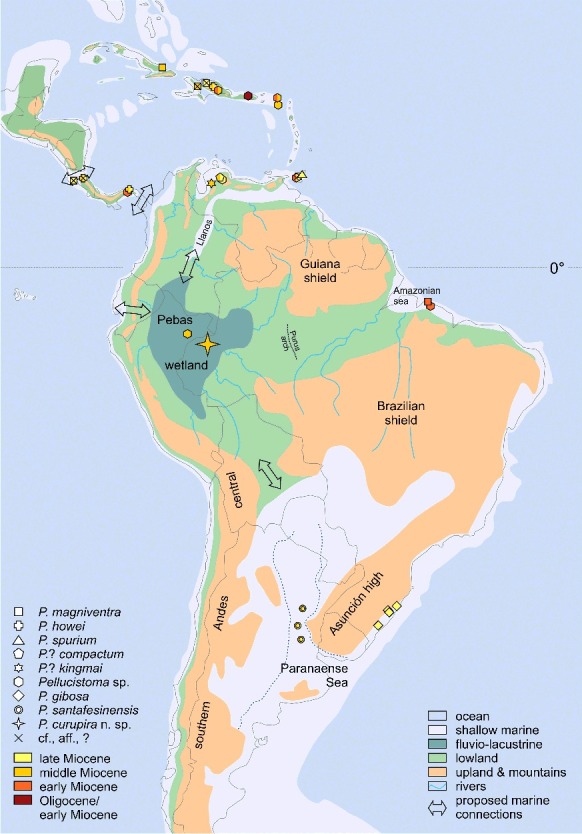
Tentative middle Miocene palaeogeography of the Caribbean realm and South America (based on Iturralde-Vinent & MacPhee 1999; Del Río 2000; Hernández *et al*. 2005; Hoorn *et al*. 2010b; Candela *et al*. 2012; extent of the Paranaense Sea probably too large (dashed blue line); compare Aceñolaza 2000; Cione *et al*. 2011; Ruskin *et al*. 2011) and Miocene records of *Pellucistoma* (the late Miocene *P. magniventra* (Florida) and *P*. aff. *spurium* (Bahamas) records are not displayed; compare Fig. 5).

Based on his extensive works on Caribbean ostracods, Bold (1974) previously outlined the dispersal of *Pellucistoma*. Bold suggested an early Miocene origin in northern South America and its subsequent spread towards the Lesser Antilles and Panama before the onset of the middle Miocene. Following our evaluations, the potentially earliest records stem from the Lares Formation of Puerto Rico (upper Oligocene–lower Miocene; Fig. 6).

Either way, early Miocene occurrences are noted from Trinidad (Brasso Formation), Panama (Culebra Formation) and Brazil (Pirabas Formation), which support a substantial dispersal event in the early Miocene (Bold 1974). Afterwards (middle Miocene), *Pellucistoma* colonized the Greater Antilles (Hispaniola, Cuba) and arrived during the late Miocene at the south-eastern coasts of North America and the Pacific coast of Costa Rica. Amazingly, it also has been spread far to the south, to southern Brazil (Pelotas basin) and north-eastern Argentina (Paraná basin) at that time. During the Pliocene, *Pellucistoma* expanded along the Atlantic coast of the USA (up to North Carolina) and settled the Gulf of Mexico (*P. magniventra*).

Its Miocene and Pliocene dispersal in the Caribbean, Gulfian and Carolinian provinces (*sensu* Cronin 1987) can be explained by repeated, short-distance transport via floating water plants between the islands and along the shoals of the American continent, following the generally west and north-west directed sea surface currents (Bold 1974; Cronin 1987; Iturralde-Vinent & MacPhee 1999). More difficult to assert is the ‘rapid’ early Miocene spread of *Pellucistoma* towards the south-east (Pirabas Formation; ∼2000 km south-east of Trinidad), directed against the modern North Brazil Current. However, during the Miocene the Panama isthmus was open, causing ocean current patterns different from today. Due to the inflow of Pacific waters through the Central American Seaway, a reversed North Brazil Current, flowing along the north-east South American coast towards the south-east, has been proposed (Prange & Schulz 2004; Heinrich & Zonneveld 2013 and references therein). As neither the Orinoco River (Díaz de Gamero 1996) nor the Amazon River (Hoorn *et al*. 2010b) were fully developed, a significant, riverine dispersal barrier (like the modern Amazon; Coimbra *et al*. 1999) was not installed during the early/middle Miocene. Tentatively, a reversed Miocene North Brazil Current facilitated the south-eastward dispersal of *Pellucistoma* along the Brazilian shelf. Comparably, the colonization of areas far in the south (Pelotas and Paraná basin) in the late Miocene could have been triggered by the south-west directed Brazil Current (Wood *et al*. 1999). At this time, the cold Falkland Current is assumed to have been less effective (Coimbra *et al*. 2009; Le Roux 2012), which enabled the proliferation of *Pellucistoma* in the Paranaense Sea. Although highly speculative, larger animals (e.g. sirenians) might have accelerated the expansion along the east coast of South America by acting as ectophoretic vectors. This hypothesis receives some support by the proposed north–south dispersal of sirenians along the eastern South American coast and their invasion of the Paranaense Sea from the south (Vélez-Juarbe *et al*. 2012).

In warmer periods of the Pleistocene, *Pellucistoma* extended further northwards on the west Atlantic coast (*P. magniventra*; e.g. Valentine 1971; Cronin 1979; Cronin & Dowsett 1990) and started to settle the Pacific coast of the USA (*P. bensoni*; Valentine 1976). In southern South America, a new species appeared (*P. elongata*; Ferrero 2009). Based on comparative morphology, *P. elongata* is much more closely related to *P. magniventra* than to the late Miocene species *P. gibosa* and *P. santafesinensis*. Thus, *P. elongata* is probably a descendant of *P. magniventra* and derives from a pre-Holocene dispersal event of the latter.

During the Holocene, *Pellucistoma* achieved its present distribution. Obviously, its latitudinal extension is limited by water temperature. While on the US Atlantic coast (∼38 ° N) the cold Labrador Current forms a thermal barrier, on the east Pacific coast the California Current confines its northward dispersal (∼34 ° N). On the south-west Atlantic coast (Argentina), the cold Falkland Current restricts its migration (*P. elongata*) further to the south. *P. elongata* has the highest latitudinal occurrence (∼42 ° S) of all *Pellucistoma* species but it is also the species with the shallowest records (at 42 ° S: littoral rock pool). As it is found in restricted bays, locally significantly warmer waters can be assumed, which permit its survival. A substantial northward migration could be hindered by the freshwater discharge of the Río de la Plata (Whatley *et al*. 1998b).

Based on the fossil and Recent records, a huge distributional gap is obvious (Fig. 5). *Pellucistoma* is missing along practically the entire Pacific coast of South America (there is only one *P. magniventra* record (one valve); Bahía de Tumaco, Colombia). Potentially this is an enormous sampling bias; however, we expect the Peru Current impeded a successful settlement of *Pellucistoma*. The Peru Current has been in existence at least since middle Miocene times (Le Roux 2012 and references therein). Its cold waters and northward-directed drift probably hampered the dispersal of *Pellucistoma* on the western coast of South America from the Miocene up to present times.

Recent *P. scrippsi* is restricted to the west coast of North and Meso America. Off Sinaloa and Nayarit (Mexico), it co-occurs with *P. magniventra*. This sympatric occurrence could be a taxonomic artefact or – speculatively – the result of a quite recent, passive, man-made dispersal event, tracing major ocean lanes from the Panama Canal towards California (Teeter 1973). Comparably, the single *P. magniventra* record on the western Colombian coast might reflect such a scattered displacement.

### The enigmatic occurrence of *Pellucistoma curupira* sp. nov. in western Amazonia: proof of marine incursions?

The spatiotemporal distribution pattern of thus far known *Pellucistoma* species can be explained by vicariant barriers (land bridges, water temperature, depth, salinity), as well as by passive transport (sea currents (drifting matter) and, possibly, phoresy). However, the crucial questions of the palaeoenvironmental implication and the provenance of our new species remain to be discussed.

Our appraisal of records of *Pellucistoma* demonstrates that it is a shallow marine taxon. No evidence of substantial deviations in habitat preferences or adaptations to non-marine environments has been reported so far. Consequently, the finding of *P. curupira* sp. nov. appears to be amongst the most solid (body fossil) biotic evidence for proposed marine incursions, affecting the centre of Amazonia in Miocene times.

#### Autecology of *Pellucistoma curupira* sp. nov

The new species, *Pellucistoma curupira* sp. nov., is evidently dwarfed in comparison to all other *Pellucistoma* species (in length ∼20–50% smaller) and very thin-shelled. There are multiple causes of size reduction or dwarfism in ostracods (temperature, oxygenation, salinity, food resources, etc.; e.g. Neale 1988; Majoran *et al*. 2000; Yin *et al*. 2001; Hunt & Roy 2006; Finston 2007; Hunt *et al*. 2010; Scheihing *et al*. 2011; Yamaguchi *et al*. 2012). Here, we cannot identify a single or several factors to be the reason for the small size of *P. curupira* sp. nov. Nevertheless, it obviously had to cope with some kind of environmental stress and lived close to its tolerance limits. Its extremely thin-shelled valves also support this theory (e.g. Frenzel & Boomer 2005 for references; compare also Vermeij & Wesselingh 2002 for marine-derived gastropods). During the preparation of this paper, Boonstra *et al*. (2015) reported *Pellucistoma* (conspecific with *P. curupira* sp. nov.; MIFR pers. obs.) from western Amazonia (middle Miocene; Nuevo Horizonte), accompanied by euryhaline foraminifers. Based on the low diversity foraminiferal assemblage and the high proportion of abnormal tests, these authors proposed a highly stressful habitat with poorly oxygenated bottom waters, close to freshwater conditions (Vonhof *et al*. 2003: <1 PSU). Hence, it seems plausible that low oxygenation (Wesselingh *et al*. 2006) and, in particular, low salinity are the main abiotic stress factors leading to the dwarfism and the poorly calcified valves of *P. curupira* sp. nov.

*Pellucistoma curupira* sp. nov. is associated in sample AM10/30 with a typical Pebasian ostracod fauna (Whatley *et al*. 1998a; Gross *et al*. 2013, 2014), totally dominated by *Cyprideis* (12 sympatric species; ∼99% of the total ostracod fauna). This ostracod genus is holoeuryhaline, able to survive hypoxic periods and especially successful in stressful settings (e.g. Gross *et al*. 2008 and references therein). The only other ostracods found in this sample are *Perissocytheridea ornellasae* (Purper, 1979), *Rhadinocytherura amazonensis* Sheppard & Bate, 1980 and *Skopaeocythere tetrakanthos* Whatley *et al*., 2000 (MG in prep.). These species are – like *P. curupira* sp. nov. – endemic for western Amazonia and supposedly of marine origin. Interestingly, they are also of minute size (∼0.30–0.35 mm in length). An adaptation to freshwater settings during the late Miocene has been demonstrated for *R. amazonensis* and *Perissocytheridea,* as well as for *Cyprideis* spp. (Gross *et al*. 2013).

Stable isotope analyses (δ^18^O, δ^13^C) performed on three *Cyprideis* species associated with *P. curupira* sp. nov. yielded very light values (Fig. 4). Such depleted δ^18^O and δ^13^C ratios are indicative of a freshwater system (Leng & Marshall 2004). Our results are consistent with previous isotopic data obtained from outcrop material (ostracods: Gross *et al*. 2013). Earlier O/C-isotopic investigations (mainly molluscs) yielded closely comparable results (Vonhof *et al*. 2003; Kaandorp *et al*. 2006; Ramos 2006; Wesselingh *et al*. 2006).

The morphology of *P. curupira* sp. nov. (very small and extremely thin-shelled), the associated ostracod fauna (co-occurrence of further stunted ostracods of potentially marine ancestry) and our geochemical evidence, suggest that this *Pellucistoma* species – exceptionally – managed to adapt to freshwater conditions in the late middle Miocene.

#### Provenance of *Pellucistoma curupira* sp. nov

We presume: (1) the genus *Pellucistoma* originated in the Caribbean realm around the Oligo-/Miocene boundary; (2) *Pellucistoma curupira* sp. nov. is most closely related to Caribbean species; and (3) *Pellucistoma* is a shallow marine clade but adapted (*P. curupira* sp. nov.) over geological timescales (about 10 million years) to freshwater conditions in western Amazonia. Based on this, we explore possible migration scenarios:

#### (A) Migration of *Pellucistoma curupira* sp. nov. via aerial (bird) transport

Bird-mediated transport plays a certain role in ostracod dispersal. In such a case, *Pellucistoma* (and other small aquatic invertebrates, e.g. foraminifers) could have entered western Amazonia without aquatic connections (neither fluvial nor marine). By considering the palaeobiogeographical distribution of *Pellucistoma*, there are two potential sources (Fig. 6): the Caribbean and the Amazonian Sea. Due to the close relationship of *P. curupira* sp. nov. to Caribbean species and half the distance to travel, the first source seems more likely. Although less well developed than today, north–south bird migration was already in existence during the Miocene (Tambussi & Degrange 2013).

Nevertheless, *Pellucistoma* is not predisposed for aerial dispersal, and successful transfer as well as ‘ad hoc’ colonization of new (freshwater) habitats appears to be demanding. (Note: such a mode of migration is much more conceivable for the eurypotent *Cyprideis*. This ostracod achieved evidently a ‘habitat monopoly’ in the Miocene of western Amazonia, possibly causing additional biotic stress for other, rather stenopotent arrivals like *Pellucistoma*). However, a multitude (over millions of years) of short-distance transport ‘accidents’ through a patchy structured wetland is possible, enabling a stepwise, long-distance spread and gradual adaptation to freshwater. Even if this scenario applies for small invertebrates, it is hardly appropriate for larger, marine-derived vertebrates occurring in Amazonia (e.g. Lovejoy *et al*. 2006; Boonstra *et al*. 2015 and references therein).

#### (B) Migration of *Pellucistoma curupira* sp. nov. through marine incursions

Several marine pathways have been proposed to have linked western Amazonia with the sea during the Miocene (for comprehensive discussions see e.g. Nuttall 1990; Rebata *et al*. 2006; Wesselingh & Salo 2006; Hovikoski *et al*. 2010). Such connections have been envisaged towards the north (Caribbean Sea), the east (Amazonian Sea), the south (Paranaense Sea) and the west (Pacific, southern Ecuador).

Concerning *Pellucistoma*, we have no evidence for an eastern Pacific source, which corroborates low faunistic affinities in the mollusc record (Wesselingh & Salo 2006). *Pellucistoma* occurs in the Pirabas Formation (early Miocene) and the Amazonian Sea could be a potential source. Nevertheless, marine incursions, originating from the eastern Brazilian Atlantic, are unlikely since the ‘Purus arch’ formed a significant watershed at least until the late Miocene (e.g. Figueiredo *et al*. 2009; Hoorn *et al*. 2010b; Latrubesse *et al*. 2010; Dino *et al*. 2012).

Relations between aquatic biota of the Paranaense Sea and the Pebas system are poor (e.g. Marengo 2000; Hulka *et al*. 2006; Wesselingh & Salo 2006; Nicolaidis & Coimbra 2008). Similarly, *P. curupira* sp. nov. is not closer related to the species of the Paraná and Pelotas basins, for which we already suggested migration along the eastern coast of South America. Potential marine connections with the Paranaense Sea are dated to the late Miocene (Hovikoski *et al*. 2007; Uba *et al*. 2009). Thus, *P. curupira* sp. nov. (late middle Miocene) pre-dates the southern South American species and immigration from the south is improbable.

Most authors agree with a linkage between the early–middle Miocene Pebas wetland and the Caribbean Sea through corridors in the Llanos basin (e.g. Nuttall 1990; Hoorn *et al*. 1995; Anderson *et al*. 2006; Lovejoy *et al*. 2006; Wesselingh & Macsotay 2006). Morphological affinities of *P. curupira* sp. nov. to the Caribbean also support this claim. Hence, a Caribbean–Llanos–Pebas-dispersal pathway is most plausible; however, fossil evidence is pending for *Pellucistoma* in the Llanos region.

Marine incursions, deriving from the Caribbean realm, have been assumed to affect western Amazonia's environments and biota (e.g. Boonstra *et al*. 2015). At first glance, our *Pellucistoma* record is a profound confirmation of such far-reaching marine influences, as it could have followed the ingressions. But, as argued above, this record does not confirm the influx of marine waters. To clarify, we do not reject the possibility of marine incursion throughout the entire Miocene history of western Amazonia. For instance, the probably slightly older middle Miocene *Pellucistoma* records from oligo-/mesohaline layers of Nuevo Horizonte (Boonstra *et al*. 2015) could be interpreted to mirror an incursion-related immigration as well as the stepwise freshwater adaptation of *P. curupira* sp. nov. Nevertheless, in our case direct marine connections are not mandatory.

#### **(C) Migration of *Pellucistoma curupira* sp. nov. through aquatic pathways.**

 The Caribbean-derived *Pellucistoma* probably reached the Llanos Basin (like other marine biota) during sporadic marine incursions in the early/middle Miocene (e.g. Wesselingh & Macsotay 2006; Jiménez & Hammen 2007; Bayona *et al*. 2007; Gomez *et al*. 2009; Boonstra *et al*. 2015 and references therein). Larger animals of marine ancestry (e.g. fishes, dolphins, manatees; Lovejoy *et al*. 2006; Lundberg *et al*. 2010; Bianucci *et al*. 2013) were able to migrate actively into the Pebas system via fluvial pathways. Smaller biota (e.g. ostracods, foraminifers, mollusc larvae) could have entered this ‘mega-wetland’ actively too but, more likely, could have been freighted ectophoretically and gradually developed freshwater tolerance over long timescales (e.g. *P. curupira* sp. nov.). Thus, “it would not seem to be necessary for the connection between the sea and the heart of the basin to be direct at any one time. A series of lakes continually splitting and merging with each other, or perhaps becoming reconnected by streams, would enable taxa to progress gradually from one area to another.” (Nuttall 1990, p. 351; compare Lundberg *et al*. 2010).

Based on our investigations and the data available, we favour hypothesis (C). However, we cannot explicitly reject hypotheses (A) and (B), which might also contribute to the enigmatic occurrence of *Pellucistoma curupira* sp. nov.

## Conclusions

Based on our in-depth evaluation of a new species of the ostracod genus *Pellucistoma* from the late middle Miocene of western Amazonia, we conclude that this genus is: (1) biogeographically restricted to the Americas; (2) in general a typical shallow marine clade; and (3) of Oligocene/early Miocene Caribbean origin. We assume that *Pellucistoma* entered the Llanos Basin during the early Miocene, migrated into the fluvio-lacustrine Pebas mega-wetland by phoresy through aquatic (fluvial) connections and adapted to freshwater conditions. Our finding emphasizes again that palaeoenvironmental interpretations based on a straightforward application of uniformitarian principles are problematical for the endemic biota of western Amazonia (Wesselingh 2006; Gross *et al*. 2013). Thus we conclude that this record of *Pellucistoma* is not evidence for marine incursions.

## Supplementary Material

SM_1.xlsx

SM_2.kml
